# 

**Published:** 1892-07-16

**Authors:** 


					ii,p,Rice twopence.
i? Vo1- XII.?July 16th, 1892
tie General Post Office as a Newspaper for
rt fusion in the United Kingdom and Abroad.]
WITH EXTRA NURSING SUPPLEMEI
Fer Annum, 8s. 6d., post free.
Monthly Farts, 8d.; post free, 8d.
Ditto per Annum, 8s., post free.
No.
303.
Vol. XII.] Saturday,
July 16th, 1892.
[Twopence.

				

